# Rapidly Progressing Autoimmune Hemolytic Anemia in a Pediatric Patient With COVID-19

**DOI:** 10.7759/cureus.45633

**Published:** 2023-09-20

**Authors:** Julia E Kononowicz, Mohammed Farhan Ali, William Palko, Sean Pyper, Nisha Agasthya

**Affiliations:** 1 Department of Pediatrics, University of Kansas School of Medicine, Wichita, USA; 2 Department of Pediatrics, Division of Nephrology, Children's Mercy, Wichita, USA; 3 Department of Pathology, Wesley Medical Center, Wichita, USA; 4 Department of Pediatrics, Division of Critical Care Medicine, Wesley Medical Center, Wichita, USA

**Keywords:** covid-19, corticosteroid treatment, rituximab therapy, pediatric case, mixed autoimmune hemolytic anemia

## Abstract

SARS-CoV-2 is a novel virus that is known to have a predilection for complications associated with the respiratory system. Although COVID-19 has a wide spectrum of manifestations, the pathophysiology of severe illness remains poorly understood but is thought to be associated with fulminant cytokine release. While severe complications secondary to COVID-19 in the pediatric population are considered rare, they do happen. Children with and without comorbidities have required intensive care unit admissions for respiratory distress and, more notably, multisystem inflammatory syndrome in children (MIS-C). While MIS-C is associated with hematologic complications, such as thrombocytopenia and coagulopathies, it is not associated with blood hemolysis. In this report, we describe a case of a 23-month-old previously healthy female, who presented with lethargy and positive COVID-19 PCR status. This case illustrates the rapid and fatal sequela caused by autoimmune hemolytic anemia (AIHA) from COVID-19. It stresses the importance of thorough workup and management of AIHA secondary to COVID-19 illness. Currently, there is limited understanding of AIHA from COVID-19 illness in children. Our aim is to describe this rare complication of COVID-19 illness in pediatric patients and discuss the best practices to manage it.

## Introduction

SARS-CoV-2 is the virus that has caused an unprecedented pandemic, with a high mortality of over six million deaths [1]. SARS-CoV-2, also known as COVID-19, has been closely studied and documented throughout the pandemic to better understand the pathophysiology, leading to frequently encountered complications of this virus, such as pneumonia, respiratory failure and, more recently documented, severe coagulopathies. While the underlying pathophysiology continues to be studied and researched, current evidence suggests a hyperinflammatory syndrome causing a fulminant cytokine storm that leads to increased disease severity with poor prognosis [2].

There has been a growing number of COVID-19-associated autoimmune hemolytic anemias (AIHAs) in patients with severe comorbidities, but few documented cases of immune-competent hosts with severe AIHA [3-8]. Current reported COVID-19-related mortality rate is 11% in adults and 4% in children [6]. We, herein, report a case of a previously healthy, 23-month-old female who presented with severe mixed-type AIHA in the setting of COVID-19.

## Case presentation

A 23-month-old, previously healthy female, weighing 13 kg, presented with two days of progressive fatigue, somnolence, and pallor with mild symptoms of upper respiratory tract illness and fever. There were no sick contacts at home. She was brought to our Emergency Department (ED) by her parents where she was noted to be ill-appearing. Her initial vital signs included a heart rate of 130 beats/minute, respiratory rate of 35 breaths/minute, oxygen saturation of 100%, blood pressure of 84/40 mmHG, and temperature of 35.6 C. On exam, she had notable pallor and was difficult to rouse. She was lethargic with minimal response to painful stimuli. Notable physical exam findings included conjunctival pallor, mild tachypnea, delayed capillary refill of 5 s, and poor muscular tone. She was noted to be profoundly hypoglycemic with serum glucose of 30 mg/dL on point-of-care testing for which she received intramuscular glucagon.

Table 1 illustrates her notable laboratory findings on complete blood count (CBC) concerning a hemolytic process in the setting of acute infection; particularly concerning was her leukocytosis with a white blood cell (WBC) count of 48.8 K/mL, severe macrocytic anemia with a hemoglobin (Hgb) of 1.9 g/dL, and mean corpuscular volume (MCV) of 130.6 fl. Table 2 shows her comprehensive metabolic panel (CMP), significant for severe metabolic acidosis with a bicarbonate of 5 mmol/L and azotemia 42 mg/dL. Hemoglobin was repeated and showed to have decreased to 1.5 mg/dL. CT brain and FAST exam showed no evidence of bleeding or traumatic injuries. Her chest x-ray was unremarkable. Blood and urine cultures resulted negative at the final.

**Table 1:** Complete Blood Count with Differential (CBC)

	Results	Normal Range
WBC (K/cumm)	49.8	5.0-18.0
RBC (K/cumm)	0.36	4.00-6.00
Hemoglobin (g/dL)	1.9	11.0-13.5
MCV (fl)	130.6	80-100
MCH (pg)	52.8	25.0-31.0
MCHC (g/dL)	40.4	32.0-37.9
RDW (%)	Too low to detect	11.0-15.6
Plt count (K/cumm)	344	150-400
MPV (fl)	10.4	7.9-9.5
Nucleated RBC %	4.2	0.0-0.0/100 WBC
Polychromasia	Noted	None
Spherocytes	Noted	None

**Table 2:** Comprehensive Metabolic Panel (CMP)

	Results	Normal Range
Sodium (mmol/L)	146	135-148
Potassium (mmol/L)	5.3	3.5-5.3
Chloride (mmol/L)	105	98-110
Carbon dioxide (mmol/L)	5	18-25
Anion Gap (mmol/L)	36	5-15
BUN (mg/dL)	42	7-20
Creatinine (mg/dL)	0.83	0.20-0.80
Glucose (mg/dL)	254	70-99
POC Glucose (mg/dL)	30	70-99
Calcium (mg/dL)	8.4	8.5-10.1
Total Bilirubin (mg/dL)	2.3	0.0-1.0
AST (Units/L)	69	16-69
ALT (Units/L)	29	<66
Total Alk Phosphatase (IU/L)	225	81-629
Serum Total Protein (g/dL)	6.6	5.7-8.0
Albumin (g/dL)	3.5	3.4-5.0

Upon transfer to the pediatric intensive care unit (PICU), she underwent emergent tracheal intubation due to her encephalopathy and inability to protect her airway, and central venous access was obtained in the right femoral vein under local anesthesia.

**Figure 1:**
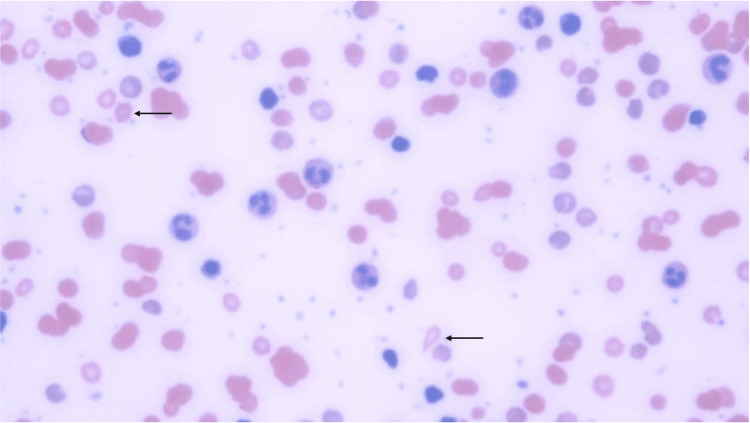
Arrows Indicate Spherocytes Indicative of Hemolytic Anemia in Peripheral Smear

Her respiratory pathogen polymerase chain reaction (PCR) panel was positive for SARS-CoV-2. Peripheral smear was negative for blasts or other malignant cells. Figure 1 shows a peripheral smear with spherocytes indicated by arrows. Serology testing for HIV and Hepatitis A, B, and C were also negative. The direct antiglobulin test (DAT) was strongly positive for IgG and weakly positive for anti-C3, indicating a predominantly warm and minor component of cold AIHA. A cold agglutinin study revealed 1:32 titer.

**Figure 2:**
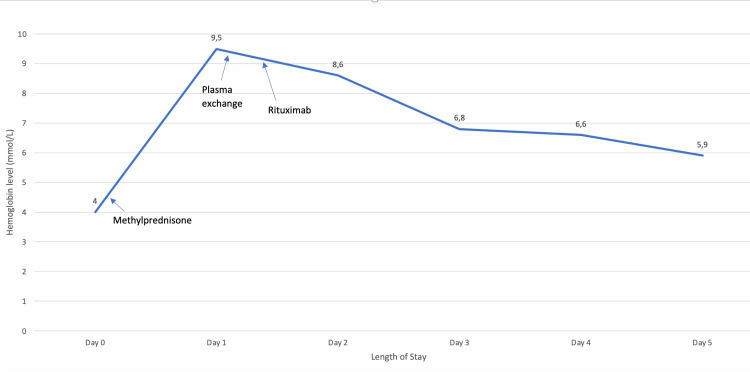
Median Hemoglobin Trend Throughout Hospital Course Following Treatment with Wethylprednisolone, Plasma Exchange, and Rituximab

Following the results of DAT in the setting of severe anemia, treatment with high-dose methylprednisolone (30mg/kg), intravenous immunoglobulin (IVIG), and packed red blood cell (RBC) transfusion was initiated. However, she developed significant hemodynamic decompensation due to ongoing hemolysis, causing low cardiac output, and experienced cardiopulmonary arrest upon initiation of IVIG with pulseless electrical activity (PEA) requiring 6 min of cardiopulmonary resuscitation (CPR) and one dose of epinephrine for return of spontaneous circulation (ROSC). IVIG infusion was stopped, and PRBC transfusion was completed over several hours, while her hemodynamics were supported with norepinephrine and epinephrine infusions. However, she continued to have profound cardiovascular instability with two more events of cardiac arrest with bradycardia lasting 5 min each. Due to ongoing metabolic acidosis and acute renal failure, she was started on continuous veno-venous hemodiafiltration (CVVHDF) with blood prime on hospital day two. A manual plasma exchange was performed on hospital day two, and rituximab (375 mg/m^2^) was administered after the completion of the plasma exchange. Figure 2 shows the median hemoglobin trend following the above-noted medical interventions. She received a total of 11 PRBC transfusions during the admission. Between hospital days three to five, her clinical parameters improved significantly with the normalization of electrolytes, and she was weaned off vasopressors by hospital day five. However, her neurologic exam remained concerning, initially with abnormal posturing, while remaining unresponsive and comatose for the entirety of admission with concern for severe neurologic injury from severe anemia and anoxic brain injury. Two brain viability tests were performed 12 hours apart on hospital day six, which were consistent with brain death and the patient died. An autopsy was not performed as per the parent’s wishes.

## Discussion

Among patients with SARS-CoV-2 infection, pneumonia, respiratory failure, and acute respiratory distress syndrome are commonly encountered complications in the adult and pediatric population [2]. Multisystem inflammatory syndrome in children (MIS-C) is an emerging and severe complication of COVID-19 that is being closely studied to formulate evidence-based guidelines for workup [9]. Complication rates associated with COVID-19 without severe comorbidities in the pediatric population are estimated to be around 1.1% and the increase in incidence with associated comorbidities to almost 7% [10]. There are few documented studies that surround hematologic complications related to this novel virus. While anemia and thrombocytopenia occur mainly in forms of severe disease and in MIS-C, they occur in asymptomatic and mildly symptomatic patients [11]. AIHA specifically is known to be a very rare illness, affecting approximately 0.2/100,000 in the 11-20 years age group, with classification as warm (65%), cold (30%), and mixed (5%) based on the thermal amplitude of autoantibodies [12].

AIHA is an acquired autoimmune disorder that develops when autoantibodies develop against self-antigens on the RBCs, which leads to their destruction by the reticuloendothelial system or complement-mediated cell destruction. Cytomegalovirus (CMV), Epstein-Barr virus (EBV), Coxsackie virus, Parvovirus B19, Hepatitis C virus (HCV), mycoplasma, and several concurrent lymphoproliferative disorders can provoke AIHA. The exact mechanism behind this viral-induced cell destruction is unknown, but in most of the literature, it is attributed to molecular mimicry, which leads to self-reactive lymphocytes and unresponsiveness to self-antigens [7]. Postulated mechanisms include the creation of cryptic antigens causing alteration in antigen presentation from the proinflammatory state triggered by the SARS-CoV-2 virus. This can stimulate T and B lymphocytes to eventually produce autoantibodies against those antigens [13].

Diagnosis of AIHA is based on evidence of hemolytic anemia, consisting of anemia, jaundice, splenomegaly, reticulocytosis, raised serum bilirubin, and a positive DAT [14]. DAT detects the presence of antibodies or complement on the RBC surface. Studies in adult patients with COVID-19 illness have shown positive DAT in 44-46% of patients [15,16]. Mixed AIHA is usually diagnosed when monospecific DAT is positive for warm IgG autoantibody and C3 and cold agglutinins with thermal amplitude >30°C. Positive DAT is the hallmark of immune hemolytic anemia, in approximately 95% of cases [12]. Although rare, SARS‐CoV‐2 infection has now been established as a secondary cause of AIHA based on cases reported in the literature [6].

In our case, we discuss the findings of mixed-type AIHA in a pediatric patient, which is unique in that it differs from the average age group of patients presented with COVID-19-induced AIHA, including mainly the adult population, and our patient had no underlying malignant or lymphoproliferative disorders. To the best of our knowledge, our patient did not have any predisposing conditions or risk factors. Furthermore, in adults, immune-mediated cytopenia seems to be more common in the moderate-to-severe respiratory form of COVID-19, while affected children presented with minimal to no respiratory symptoms, such as our patient [11]. While AIHA in COVID-19 patients is already found to be a rare occurrence, the rapid progression and severity of our patient’s illness, a previously healthy individual, is an incredibly rare phenomenon and warns future providers about rapid decompensation. 

As this disorder is so precarious and unpredictable, it is important to quickly identify AIHA and initiate therapy as quickly as possible. The warm component of mixed AIHA is particularly known to cause rapid hemolysis and severe anemia. It can be fatal due to acute presentation or treatment refractoriness, leading to acute relapses that require multiple treatment modalities. 

Corticosteroid therapy and avoiding exposure to cold are the mainstay of treatment of mixed AIHA. Treating any underlying illness is necessary as well. If the response to steroids is unsatisfactory or the anemia is very severe, IVIG and/or plasmapheresis should be considered as well. Some AIHA cases may be refractory to steroids and require second-line treatment with rituximab, an anti-CD 20 monoclonal antibody targeting B-lymphocyte or splenectomy [6]. While the treatment guidelines for AIHA are well-documented, blood transfusion guidelines remain limited [17].

Due to the presence of pan-reacting autoantibodies, identifying cross-matched blood products may not be possible. If severe anemia is present, clinicians should contact the blood bank immediately for type-specific, “least incompatible” blood products. In some patients with rapid hemolysis and life-threatening anemia, such as our patient, PRBC transfusion can be lifesaving and should not be delayed.

## Conclusions

Mixed-type AIHA is a rare phenomenon found among children, but with its high degree of variability and clinical severity, it is imperative to diagnose it early and initiate treatment rapidly. Our case illustrates that SARS-CoV-2 may itself be capable of inducing severe AIHA even in patients with no underlying predisposition, which has not been well documented in the literature. While initiating the mainstay of treatment - steroids - quickly, the clinician must be judicious about life-saving interventions, such as least-incompatible blood transfusions. Further investigation is warranted to understand poor outcomes and escalate treatment as necessary.
